# Accessibility of Naloxone in Pharmacies Registered Under the Illinois Standing Order

**DOI:** 10.5811/westjem.17979

**Published:** 2024-05-21

**Authors:** P. Quincy Moore, Kaitlin Ellis, Patricia Simmer, Mweya Waetjen, Ellen Almirol, Elizabeth Salisbury-Afshar, Mai T. Pho

**Affiliations:** * Permanente Medical Group, Oakland, California; † Kaiser Permanente Oakland Medical Center, Department of Emergency Medicine, Oakland, California; ‡ Brown University, Department of Obstetrics and Gynecology, Providence, Rhode Island; § University of Chicago, Department of Medicine, Chicago, Illinois; ∥ University of Chicago Pritzker, School of Medicine, Chicago, Illinois; ¶ University of Chicago, Chicago Center for HIV Elimination, Chicago, Illinois; # University of Wisconsin-Madison, School of Medicine and Public Health, Department of Family Medicine and Community Health, Madison, Wisconsin; ** University of Wisconsin-Madison, School of Medicine and Public Health, Department of Population Health Sciences, Madison, Wisconsin; †† University of Chicago, Department of Medicine, Section of Infectious Diseases and Global Health, Chicago, Illinois

## Abstract

**Introduction:**

To expand access to naloxone, the state of Illinois implemented a standing order allowing registered pharmacies to dispense the drug without an individual prescription. To participate under the standing order, pharmacies were required to opt in through a formal registration process. In our study we aimed to evaluate the availability and price of naloxone at registered pharmacies.

**Methods:**

This was a prospective, de-identified, cross-sectional telephone survey. Trained interviewers posed as potential customers and used a standardized script to determine the availability of naloxone between February–December, 2019. The primary outcome was defined as a pharmacy indicating it carried naloxone, currently had naloxone in stock, and was able to dispense it without an individual prescription.

**Results:**

Of 948 registered pharmacies, 886 (93.5%) were successfully contacted. Of those, 792 (83.4%) carried naloxone, 659 (74.4%) had naloxone in stock, and 472 (53.3%) allowed purchase without a prescription. Naloxone nasal spray (86.4%) was the formulation most commonly stocked. Chain pharmacies were more likely to carry naloxone (adjusted odds ratio [aOR] 3.16, 95% confidence interval [CI] 1.97–5.01, *P* < 0.01) and have naloxone in stock (aOR 2.72, 95% CI 1.76–4.20, *P* < 0.01), but no more likely to dispense it without a prescription. Pharmacies in higher population areas (aOR 0.99, 95% CI 0.99–0.99, *P* < 0.05) and rural areas adjacent to metropolitan areas (aOR 0.5, 95% CI 025–0.98, *P* < 0.05) were less likely to have naloxone available without a prescription. Associations of naloxone availability based on other urbanicity designations, overdose count, and overdose rate were not significant.

**Conclusion:**

Among pharmacies in Illinois that formally registered to dispense naloxone without a prescription, the availability of naloxone remains limited. Additional interventions may be needed to maximize the potential impact of a statewide standing order.

Population Health Research Capsule What do we already know about this issue?

*Most states offer naloxone at pharmacies without a prescription, but uptake is limited.*

What was the research question?

*Which pharmacies registered under the Illinois Naloxone Standing Order had naloxone available without a prescription?*

What was the major finding of the study?

*Only 53.3% of registered pharmacies (1/8^th^ of all Illinois pharmacies) had naloxone in stock and available without a prescription.*

How does this improve population health?

*Statewide standing orders are an important but insufficient step toward widespread naloxone possession. More effort is needed to improve participation.*

## INTRODUCTION

The rise of opioid-related overdose has had a devastating effect on communities across the United States. In 2020 alone, over 68,000 people died from opioid-related overdose, of which almost 3,000 occurred in the state of Illinois.^1^^,^^2^ The rapidly evolving drug market, with the introduction of fentanyl, fentanyl analogues, and xylazine into the illicit drug supply, has contributed to the increasing opioid overdose fatality rates, with 64% of US drug overdose deaths during May 2020–April 2021 involving illicitly manufactured fentanyl.^3^^–^^5^

In response to the opioid overdose epidemic, a multi-pronged approach has been enacted to reduce morbidity and mortality. Among these are several harm reduction strategies, including syringe service programs, infectious disease screening, drug checking (eg, fentanyl test-strip distribution), supervised consumption sites, and distribution of naloxone. Multiple studies have demonstrated naloxone’s ability to be used effectively and appropriately by people with no formal medical training.^6^ For example, Enteen et al found that of the 24% of patients who returned for naloxone refills over a six-year period, 11% of those reported using naloxone during an overdose event, with an 89% success rate of overdose reversal.^7^ Further, studies have shown that naloxone distribution does not lead to increased opioid consumption and may even lead to decreased use.^7^^,^^8^ Recognizing its safety and efficacy, the US Surgeon General issued an advisory notice in 2018 encouraging its use and availability.^9^ Despite widespread support by leading healthcare organizations and federal agencies, naloxone access remains limited, and opportunities to help individuals at risk for overdose are frequently missed.^10^^,^^11^

As of 2017, all 50 states had passed legislation expanding public access to naloxone.^12^ In addition to legislation protecting against civil, criminal, or professional liability for both prescribers and lay administrators of naloxone, some states have introduced policies to increase the accessibility of the life-saving drug. Studies have demonstrated that pharmacists are willing to provide naloxone to the public under a standing order or other similar process (Stewart et al, 2018; Nielsen et al, 2016; Green et al, 2017). To expand access to naloxone, the Illinois Department of Public Health (IDPH) implemented a statewide standing order in 2017 (Public Act 99–0480), allowing registered pharmacies to distribute naloxone to patients without an individual prescription in their name. To register under the Illinois Naloxone Standing Order, licensed pharmacies must participate in a pre-approved training and agree to report any dispensed naloxone to the Illinois Prescription Monitoring Program.^13^

Illinois is now one of 49 states that allow pharmacists to dispense naloxone without a patient-specific prescription from a clinician, 44 of which use a standing order.^14^ Despite this, studies from other states have shown limited uptake of these new protocols and wide variations in availability of naloxone at registered pharmacies.^15^^–^^22^ In this cross-sectional study we aimed to evaluate the accessibility of naloxone at pharmacies registered under the statewide standing order by determining which pharmacies reported routinely carrying naloxone, which pharmacies had naloxone currently in stock, which pharmacies were willing to dispense naloxone without a prescription, which formulations were carried, and the out-of-pocket cost of naloxone. Our primary outcome was to determine which pharmacies had naloxone available without a prescription on the day of the inquiry. We further compared pharmacies’ naloxone availability by pharmacy type (chain vs non-chain), urbanicity, population of ZIP Code, and opioid overdose rates in the pharmacies’ surrounding region. This study expands on the existing literature by using a sample that included all pharmacies that opted in to registering under the Illinois Naloxone Standing Order. We also analyzed factors that may affect the likelihood that a pharmacy had naloxone available without a prescription, which was rarely done in previous studies.

## METHODS

### Study Design

A prospective, anonymous, cross-sectional “secret-shopper” telephone survey sampling all Illinois pharmacies that had registered under the state-level standing order was performed by six trained callers. The list of pharmacies registered under the standing order was accessed on February 17, 2019 (Chicago) and May 23, 2019 (remainder of Illinois) via the IDPH Opioid Data Dashboard.^2^ The list of pharmacies, their cities, and their contact numbers were transposed from the dashboard into an Excel document (Microsoft Corp, Redmond, WA) for tracking purposes. For each pharmacy, we obtained a ZIP Code and evidence of continued operation via Google searches. If a pharmacy was found to no longer be in existence, the pharmacy was marked as unable to contact.

### Data Collection

Six study personnel (one attending physician, one resident physician, three medical students, and one master’s level research associate) underwent three hours of training consisting of reviewing the call script, discussing the logic behind each question, discussing specific language to use, and conducting at least three pilot calls to pharmacies not included in the study sample. Pilot calls were debriefed as a group.

The callers posed as potential customers and used a standardized script to ask targeted questions. Callers followed automated prompts or requested to be connected to the pharmacy. Callers spoke with whichever pharmacy staff first answered the call and continued to use the script if the call was transferred to other pharmacy staff. If placed on hold, the caller waited up to 10 minutes before terminating the call. If the call was interrupted or the pharmacy was unreachable on the initial attempt, the pharmacy was contacted up to two additional times. If a pharmacy was unreachable three times, it was considered inactive and not included in our analyses. Calls were completed from February–December 2019. Data was collected either directly into REDCap 9.5.35 LTS (Research Data Capture hosted at University of Chicago Medicine) or into Microsoft Excel and later transposed into REDCap.

The script for the calls was created using an iterative process by the group of investigators. We designed the script to address the study questions while maintaining the appearance of a lay caller. The generic name of the medication (naloxone) was used initially. If staff seemed uncertain of the medication in question, the brand name of Narcan was used after first repeating the generic name. See Appendix 1 for the script for the secret-shopper telephone survey of pharmacies that are registered under the Illinois Naloxone Standing Order.

### Measures

We collected characteristics for each pharmacy based on pharmacy type, urbanicity, population of pharmacy ZIP Code, and the overdose rate in the pharmacy ZIP Code. Pharmacies were classified as “chain” if they had four or more locations under shared ownership, and “non-chain” if they had fewer than four locations.^15^^,^^16^ We defined urbanicity using the US Department of Agriculture 2013 Rural-Urban Continuum Codes (RUCC) that assign counties a score on a scale of 1‐9 based on county population size and adjacency to a metropolitan area.^17^ As commonly practiced elsewhere in the literature, we divided this continuum into three groups: 1) urban; 2) rural adjacent to a metropolitan area; and 3) rural and nonadjacent to a metropolitan area.

We used ZIP Codes corresponding to each pharmacy to analyze the data using overdose rates and population. Number of combined fatal and non-fatal opioid-related overdose events in 2018 by ZIP Code was obtained from the IDPH Opioid Dashboard.^2^ We obtained population by ZIP Code for 2018 from the US Census Bureau.^18^ Using the population size and the number of overdoses, we calculated a 2018 rate of combined fatal and non-fatal opioid-related overdose per 10,000 people for each ZIP Code in our sample.

#### Statistical Analyses

We performed bivariate analyses to determine whether differences in naloxone availability on the day of the call were significantly different based on the following covariates: pharmacy type; urbanicity using RUCC code; population of pharmacy ZIP Code; and the 2018 overdose count and overdose rate per 10,000 residents in the pharmacy ZIP Code. We analyzed data using STATA MP v17 statistical software release 15 (StataCorp, LLC, College Station, TX). This study was reviewed by the University of Chicago Investigational Review Board and determined to be exempt from review.

## RESULTS

**Figure 1.**
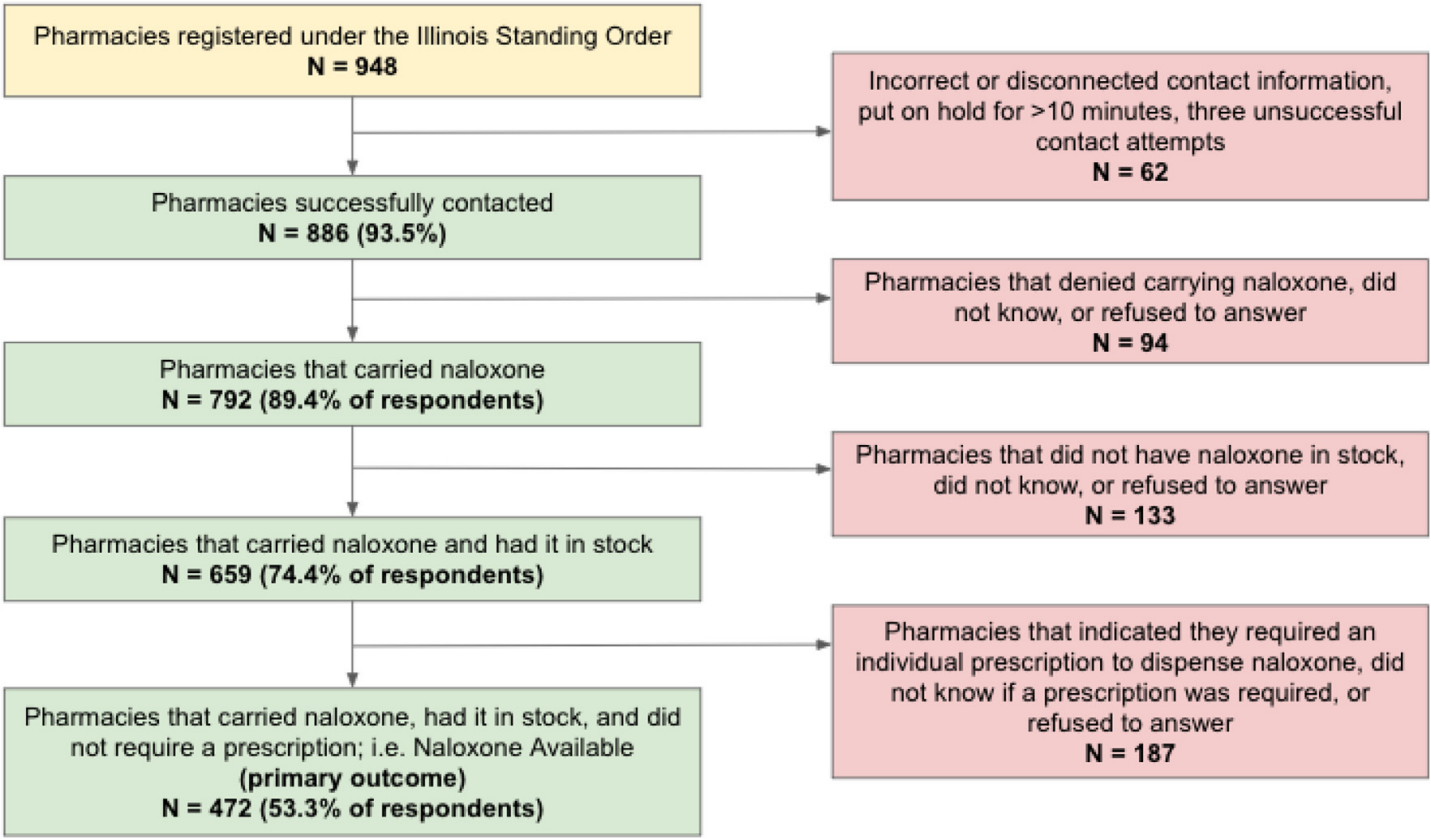
Availability of naloxone and need for a prescription in Illinois pharmacies registered under the Illinois Naloxone Standing Order.

We identified 948 pharmacies registered under the Illinois Naloxone Standing Order and successfully contacted 886 (93.5%) (Figure 1). Of the 886 pharmacies that were successfully contacted, 806 (91.0%) were chain pharmacies and 80 (9.0%) were non-chain. Of the 886 contacted pharmacies, 807 (91.1%) were located in urban ZIP Codes, 57 (6.4%) in rural ZIP Codes adjacent to a metropolitan area, and 22 (2.5%) in rural ZIP Codes that were nonadjacent to a metropolitan area. Additionally, of the contacted pharmacies, 792 (89.4%) reported carrying naloxone, with 659 (74.4%) reporting the medication to be in stock at the time of the call, and 472 (53.3%) responding that the caller did not need a prescription from a doctor to purchase the naloxone. The 472 pharmacies (53.3%) that carried naloxone, had naloxone in stock, and offered naloxone without requiring a prescription were considered positive for the primary outcome. Pharmacy characteristics are summarized in Table 1.

**Table 1.** Pharmacy type, urbanicity, and naloxone availability of pharmacies registered under the Illinois Naloxone Standing Order that were successfully contacted (n = 886).

	Successfully contacted, n = 886 (Col %)	Carry Naloxone n = 792 (Row %)	Carry Naloxone, in stock n = 659 (Row %)	Naloxone available without a Rx, n = 472 (Row %)
Pharmacy type				
Chain (CVS, Walgreens)	806 (91.0%)	728 (90.3%)	611 (83.9%)	432 (70.7%)
Non-chain (Independent)	80 (9.0%)	64 (80.0%)	48 (75.0%)	40 (83.3%)
RUCC				
Urban	807 (91.1%)	720 (89.2%)	599 (83.2%)	433 (72.2%)
Rural adjacent to a metropolitan area	57 (6.4%)	52 (91.2%)	43 (82.7%)	28 (65.1%)
Rural and nonadjacent to a metropolitan area	22 (2.5%)	20 (90.9%)	17 (85.0%)	11 (64.7%)

*Rx*, prescription; *RUCC*, Rural-Urban Continuum Codes.

**Figure 2.**
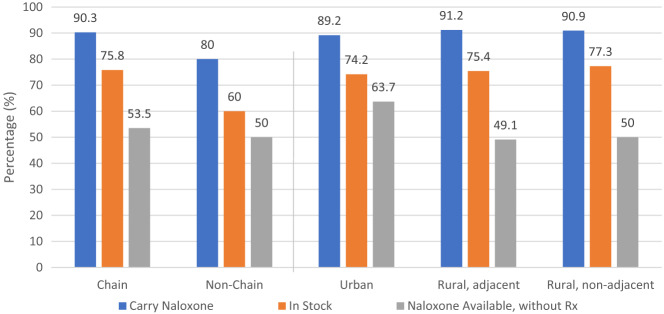
Pharmacy type, county urbanicity, and naloxone availability of pharmacies registered under the Illinois Naloxone Standing Order that were successfully contacted. *Rx*, prescription.

Figure 2 displays the cascade of naloxone availability by pharmacy type and RUCC. Pharmacies in urban RUCC codes had the highest naloxone availability without a prescription (63.7%). A larger proportion of chain pharmacies carried naloxone (90.3%) compared to non-chain pharmacies (80.0%) (*P* < 0.01). Of the 772 pharmacies that stocked naloxone and provided a response to the type of naloxone, 624 (78.8%) carried naloxone nasal spray (see Table 2).

**Table 2.** Of those who carry naloxone, available formulations of naloxone and median price.

Naloxone types	N = 722 (%)	Median price [IQR]
Naloxone nasal spray	624 (86.4)	$135.99 [$89.99, $4,500]
IM vials	71 (9.8)	$39.50 [$21.99. $239.00]
Naloxone autoinjector	27 (3.8)	$4,000 [$399.59, $6,000.00]

*IQR*, interquartile range; *IM*, intramuscular.

In the adjusted analyses, we found that chain pharmacies had greater odds of carrying naloxone (adjusted odds ratio [aOR] 3.16, 95% confidence interval [CI] 1.97–5.01, *P* < 0.01) and having naloxone in stock (aOR 2.72, 95% CI 1.76–4.20, *P* < 0.01) compared to non-chain pharmacies (Table 3). However, there were no differences between pharmacy type and naloxone availability without a prescription. With regard to RUCC, rural adjacent to a metro area had lower odds compared to urban areas of providing naloxone without a prescription (aOR 0.50, 95% CI 0.25–0.98, *P* = 0.05). We also observed that more densely populated ZIP Codes were less likely to have naloxone available without a prescription (aOR 0.99, 0.99–0.99, *P* < 0.01). Neither overdose (OD) count nor OD rate were associated with naloxone availability.

**Table 3.** Association between predictors and carry naloxone, in stock, and no prescription needed.

	Carry Naloxone		In stock		No Rx	
	aOR (95% CI)	*P-value*	aOR (95% CI)	*P-value*	aOR (95%CI)	*P-value*
Pharmacy type						
Non-chain	*Ref*		*Ref*		*Ref*	
Chain	**3.16 (1.97, 5.01)**	**<0.01**	**2.72 (1.76, 4.20)**	**<0.01**	0.45 (0.20, 1.00)	0.05
RUCC						
Urban	*Ref*		*Ref*		*Ref*	
Rural adjacent to a metro area	1.77 (0.79, 3.98)	0.17	1.27 (0.69, 2.36)	0.44	**0.50 (0.25, 0.98)**	**0.05**
Rural, nonadjacent to a metro area	1.16 (0.41, 3.30)	0.78	1.15 (0.47, 2.82)	0.75	0.48 (0.17, 1.36)	0.17
Population by ZIP Code	1.00 (0.99, 1.00)	0.61	1.00 (0.99, 1.00)	0.40	**0.99 (0.99, 0.99)**	**0.003**
OD count	1.00 (0.99, 1.00)	0.74	1.00 (0.99, 1.00)	0.42	0.99 (0.98, 1.00)	0.09
OD rate	0.99 (0.98, 1.00)	0.16	0.99 (0.99, 1.00)	0.36	0.99 (0.98, 1.00)	0.27

**Bold**, *P* ≤ 0.05; Adjusted analyses include controlling for pharmacy type, RUCC, and population by ZIP Code.

*Rx*, prescription; *aOR*, adjusted odds ratio; *CI,* confidence interval; *RUCC*, Rural-Urban Continuum Codes; *OD*, overdose.

## DISCUSSION

Standing orders are an important step toward reducing opioid-related mortality, but our findings suggest this legislation has not had the desired effect in state residents’ access to naloxone. In 2019, two years after the implementation of the order, there was an average of 3,861 licensed pharmacies statewide.^19^ Of these, only 948 (24.6%) were registered under the standing order at the time of our study. We successfully contacted 91% of the registered pharmacies and found that just over half (53.3%) had naloxone available on the day of contact and appropriately offered it without requiring a prescription. Given that all pharmacies on our contact list underwent pre-approved training to register with IDPH as a naloxone distribution site under the standing order, our findings indicate there is substantial room for improvement.

Studies from other states with comparable statewide naloxone access policies have shown limited uptake with wide variations in availability of naloxone. Across California, Texas, Pennsylvania, Massachusetts, and New York, the proportion of pharmacies that had naloxone in stock ranged from 23.5–70%, with some variation based on state and the specific sample of pharmacies studied.^20^^–^^24^ Few studies have analyzed specific characteristics that may affect an individual pharmacy’s likelihood of having naloxone available.^22^^,^^25^ In Pennsylvania, Graves et al found that chain pharmacies were more likely to carry naloxone, but OD rate and urbanicity did not influence naloxone availability.^22^ In Indiana, Meyerson et al found that chain pharmacies, pharmacies with more than one full-time pharmacist, and those where pharmacists had received naloxone-related continuing education were associated with increased likelihood of stocking naloxone.^25^

A systematic review of the topic found that a heterogeneous group of 30 studies had wide-ranging findings, but overall one-third of pharmacies audited did not carry naloxone and almost half did not offer naloxone without a prescription.^26^ While previous studies have explored the availability of naloxone under a standing order in different states, analysis of factors that may contribute to the likelihood that a pharmacy has naloxone available without a prescription remains limited. Our study is also unique for its high response rate as well as our use of a sample including all pharmacies that opted in to formalized training and registration under the standing order.

Improved access to naloxone through community pharmacies may come through multiple approaches. First, with less than a quarter of pharmacies registered, our findings highlight the need for more widespread participation in the Illinois Naloxone Standing Order. It appears that the public good and the financial incentives attached to increased dispensing of naloxone are insufficient to incentivize pharmacies to take the steps necessary to register under the standing order. Of note, Illinois Medicaid plans are required to cover at least one formulation of naloxone, with the intranasal formulation the most commonly covered formulation. Illinois Medicaid does not charge a copay for receipt of naloxone. Additional incentives may be necessary to mobilize greater pharmacy participation statewide.

Rural areas appeared to have particularly poor access to naloxone through community pharmacies. While 11.5% of Illinois residents live in rural areas, we found that only 22 (2.3%) of the pharmacies registered under the standing order were in rural areas.^27^ While there was no significant difference in the primary outcome in rural vs urban pharmacies, the overall paucity of registered pharmacies in rural areas highlights a lack of access that may put rural people who use drugs at higher risk of death from overdose. This may further exacerbate the disproportionate impact of the opioid crisis on rural areas.^28^^,^^29^

Of the registered pharmacies we contacted, our findings highlight specific trends that may inform efforts to improve access to naloxone. We found that chain pharmacies were more likely than non-chain pharmacies to carry naloxone and have it in stock but were no more likely to have it in stock without a prescription required. This suggests that there are policies unique to chain pharmacies that facilitate registering under the standing order and stocking naloxone, but that perhaps training for customer-facing staff has been inadequate. This led ultimately to similar outcomes to non-chain pharmacies when it came to customers seeking to purchase naloxone without a prescription. These findings have some consistency with one Pennsylvania study, which found chain pharmacies to be more likely to carry naloxone and answer questions correctly about the standing order for naloxone.^22^ Chain pharmacies may have more standardized training programs for certain staff members, maintain robust supply chains for naloxone, or have a stronger response to public pressure to contribute to reducing opioid-related deaths.

There was no statistically significant association between the number or rate of ODs in a ZIP Code and likelihood of naloxone availability. This finding suggests that there may be additional outreach or incentives necessary to encourage pharmacies in areas with the highest rates of OD to increase access to naloxone via the standing order.

Cost and available formulation may have a significant impact on how likely a customer is to obtain naloxone. In our sample, both cost and formulation were variable. The majority of pharmacies that had naloxone in stock carried the nasal naloxone spray (brand name Narcan) for an average cost of $135.99 for a two-pack. While Illinois Medicaid plans cover at least one formulation of naloxone without copay, private insurance and Medicare Part D plans have variable copay structures and formulation coverage. For uninsured individuals, those who don’t want to use their insurance to fill this medication, or those for whom naloxone is not a covered medication, the out-of-pocket cost may be a significant deterrent to obtaining naloxone. Vials of naloxone, which can be used with a needle and syringe and injected intramuscularly, or with an atomizer for nasal administration, had a lower median price of $39.50; however, only 9% of pharmacies had this formulation in stock, and the availability and cost of other necessary supplies such as syringes, intramuscular needles, and/or nasal atomizers was unclear. We do believe that some of the high prices that were reported by pharmacy staff are inaccurate and for this reason we present the median price, which we believe accurately reflects what most consumers would pay out of pocket.

Our study highlights the need for additional strategies to maximize access to naloxone. Given that rural areas are less likely to have community-based naloxone distribution (often a service offered at harm reduction/syringe service programs), this need is particularly great in rural areas.^30^^–^^32^ Future research is needed to understand whether naloxone availability in pharmacies is associated with increased utilization and, if so, how to increase availability of naloxone via standing order in retail pharmacies. Possible considerations could include the following: public education campaigns that would work to increase demand for naloxone in pharmacies, thereby encouraging pharmacies to register and stock naloxone; offering financial incentives or other public recognition for pharmacies that register for the standing order and stock naloxone formulations; and improved public health outreach and educational programs (eg, academic detailing) to increase awareness among pharmacies, pharmacists, and pharmacy staff about the purpose of and evidence base of naloxone as it relates to reducing opioid-related mortality at the community level.

Research has found that pharmacists’ discomfort dispensing naloxone to customers remains an important barrier and often results from inadequate training (Green, 2017; Thornton, 2017; Rudolph, 2018). As of November 20, 2017, only 19 states had mandated naloxone education for pharmacists (Roberts, 2019).^33^ Illinois regulation requires participating pharmacists to complete an Illinois Department of Human Services- approved training module or to “understand the Naloxone Standardized Procedures” and watch two training videos (IDPH Naloxone FAQ), but it is unclear how much of this training is passed along to staff who directly interact with customers. One study comparing training material provided by states found that while most material covered the purpose and use of naloxone as well as the standing order legislation, few provided thorough education on how to communicate this information to customers (Carpenter, 2018). Overall, while there has been an increase in naloxone dispensed across all states with expanded access policies, retail pharmacy naloxone distribution is still underused and varies state by state (Xu, 2018).

## LIMITATIONS

Our study has several limitations. We did not clarify the role of the staff member with whom we were speaking. It is possible that if we had asked to speak directly to the pharmacist, we would have obtained more accurate information; however, we felt it was most useful to mimic a more natural consumer interaction. It is possible, however, that responses would vary between staff members at an individual pharmacy. Information may also have been more accurate had we identified ourselves as academic research staff. Five of six callers had at least some medical background, but we believe that other studies could achieve the same goal in an analogous study using staff with no medical background.

We did not call pharmacies that were not listed on the IDPH website; so future research may include analysis of the percentage of total pharmacies in different regions that offer naloxone. We collected only information about out-of-pocket cost, which is likely only relevant to patients without insurance, those who don’t want to use insurance when receiving naloxone, or those without naloxone included in their pharmacy benefit. Lastly, and perhaps most relevant to future research, we recognize that availability of naloxone in retail pharmacies may not directly correlate with increased utilization by people who use drugs (PWUD). Future studies should incorporate input from PWUD to delineate preferences in sources of naloxone.

## CONCLUSION

We found that two years after implementation of the Illinois Naloxone Standing Order, only one-eighth of all pharmacies had naloxone in stock and available without a prescription. Within this group, chain pharmacies were more likely to carry naloxone and have it in stock but were no more likely to provide it without a prescription. Pharmacies in more densely populated ZIP Codes and those with a Rural-Urban Continuum Code reflecting rural areas that are adjacent to metro areas were less likely to have naloxone available without a prescription. Overdose rates in the surrounding community had no effect on naloxone availability. Our study illustrates a unique sample of all pharmacies statewide that have gone through formal training and registration under the standing order.

Increased access to naloxone in retail pharmacies in Illinois will require improved efforts related to awareness and implementation of the standing order, as well as further investigation into the reasons that a pharmacy that has gone through the process of applying to be able to use the standing order does not reliably stock naloxone and make it available without prescription. Specific attention should be given to areas where there is limited access to naloxone through community-based dispensing programs and where rates of overdose and potential for impact are highest.

## Supplementary Information




