# Supplementary material for: Accessibility of Naloxone in Pharmacies Registered Under the Illinois Standing Order
Source: West J Emerg Med. 2024 May 21;25(4):457–64. doi: 10.5811/westjem.17979 (PMC11254148; doi:10.5811/westjem.17979)
Supplement: Supplementary file 1 [file wjem-25-457-s001.docx]

**Appendix 1:** Script for secret-shopper telephone survey of pharmacies that are registered under the naloxone standing order in Illinois

Hi, I’m calling to see if you guys carry naloxone

Do you have it in stock right now?

No

(If not already addressed) Why not?

END CALL

(If not already addressed)

If I get a prescription, could you give it to me?

Do I need a prescription?

Yes

Yes

No

What kinds do you guys have in stock?

(If needed) I’ve heard it can be given either with a shot or by nose.

How much does each kind cost?

If asked whether or not insured, answer is no

Do I need to do anything to get it? Like a training?

Can I buy it even if I’m not planning on using it on myself?

END CALL

END CALL
